# Experimental and Numerical Analyses of the Failure of Prestressed Concrete Railway Sleepers

**DOI:** 10.3390/ma13071704

**Published:** 2020-04-05

**Authors:** Ramon Silva, Welington V. Silva, Jonas Yamashita de Farias, Marcos Aires A. Santos, Leonardo O. Neiva

**Affiliations:** Department of Civil and Environmental Engineering, SG-12 Building, Darcy Ribeiro Campus, University of Brasilia, Brasilia 70910-900, Brazil; welington.vital@gmail.com (W.V.S.); jonasyamashita@hotmail.com (J.Y.d.F.); marcosairess@gmail.com (M.A.A.S.); leonardo.ferrovias@gmail.com (L.O.N.)

**Keywords:** prestressed concrete sleeper, rail seat, corrosion, railway engineering, finite element analysis

## Abstract

This paper carries out the assessment of load-carrying capacity of prestressed concrete sleepers, in accordance with Brazilian Standard (ABNT NBR 11709) and AREMA Standard. In a lot of railways around the world, many prestressed concrete sleepers have failed due to Rail Seat Abrasion (RSA) and corrosion. RSA is the wear degradation underneath the rail on the surface of prestressed concrete sleepers. In this paper, a numerical study was carried out to evaluate the load-carrying capacity of the prestressed concrete sleepers, using ABAQUS software. The nonlinear using Concrete Damage Plasticity model was validated by 18 experimental results, in accordance to standards. Using the validated model, the influence of different wear depth RSA, combined with corrosion of the prestressed wires, is investigated.

## 1. Introduction

The sleeper is one of the fundamental elements of the superstructure of the railway tracks. In summary, its main functions are to support the rails, keep the axis of the track constant and transmit to the ballast the actions of the axles of the vehicles. These functions make it necessary to provide the sleeper with a high level of strength, which in general leads to great stiffness; at the same time, it is also necessary to have a certain level of elasticity, since it must be able to withstand extremely high impact forces [1,2,3]. In the early twentieth century, the first reinforced concrete sleepers imitated, in their design, the shape of wooden sleepers, consisting of a concrete block with a constant section. The results were not satisfactory, as the shocks and vibrations arising from the dynamic actions of the vehicles quickly caused cracks in the upper and central part of the sleeper; despite the steel bar placed to withstand tensile forces, they were not effective in preventing the collapse of the sleeper. Steel bars with stirrups were used to avoid shear cracks; however, it was not very efficient, due to the difficulty of manufacturing the sleeper, and cracks in the concrete contributed to corrosion of steel bars [3,4,5,6,7].

In order to overcome this deficiency, with a bigger nominal concrete cover, to avoid corrosion of the reinforcement concrete, it was applied in concrete the corrosion inhibitors. Corrosion inhibitors are useful in cases where it is not possible to achieve full concrete cover on the reinforcement; however, they raise the price of the sleeper [8,9,10,11].

Thus, with the development of prestressed concrete, the possibility arose to combat cracks in concrete, giving rise to a new stage in the design of concrete sleepers. Prestressed concrete sleepers used in railways emerged historically from World War II, and recently there was an increase in their use due to their technical superiority, in order to replace wooden sleepers [11].

The purpose of the prestressed force on the rebars is that the stress applied to the steel wires allows the material to remain compressed, even under bending, thus avoiding the opening of tensile cracks. Recently, the need for sleepers’ replacement in old railroads has been increasing, as well as the average increase of the speed guide provided by increasingly modern trains, which economically justifies the large amount of studies on this theme around the world [5,12,13].

The sleepers are subjected to cyclic loads during their whole life; the materials that comprise them are subjected to an intense process of fatigue. It is desirable that the sleeper is free of cracks under dynamic loading, because if cracks occur, there is a great increase in stresses in the prestressing reinforcement and an increase of up to 50% in the transfer length [14,15,16]. Therefore, designing a prestressed sleeper is a good solution to avoid problems with simple concrete [1,15,17,18].

Thus, this paper aims to evaluate the mechanical behavior of prestressed concrete sleepers, in accordance with the Brazilian and American standards and the common pathologies they are submitted to over their design life. A comparison is made between the results obtained numerically by the software ABAQUS and the experimental curves obtained through the tests, as recommended by ABNT NBR 11709 [19] and AREMA [20]. Eighteen experimental tests in sleepers were carried out, in order to validate the nonlinear numerical model, using ABAQUS [18]. In addition, using the validated numerical model, the influence of the combined effect of rail seat abrasion and corrosion in the load-carrying capacity of the sleepers was evaluated [4,10,21,22,23].

## 2. Experimental Investigations

### 2.1. Details of Prestressed Sleeper

Eighteen sleepers were taken from the Brazilian railway and were tested in in the Infrastructure Laboratory—INFRALAB and in the Structures Laboratory—LABEST of the University of Brasilia, using the Brazilian Standard (ABNT NBR, 11709) [19].

**Figure 1:**
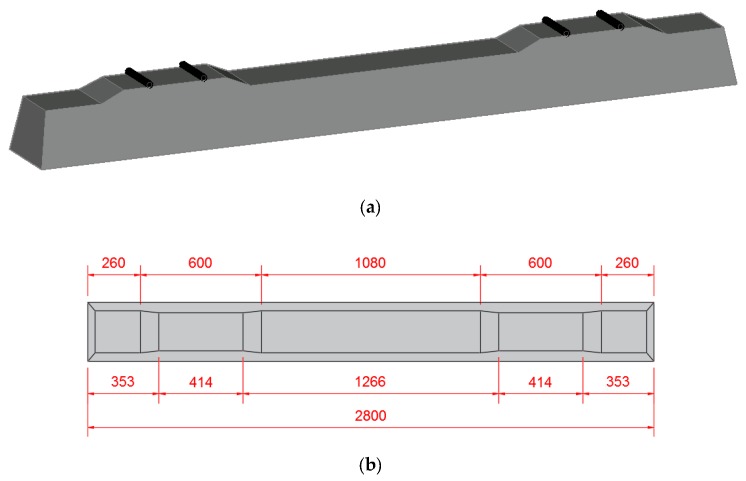

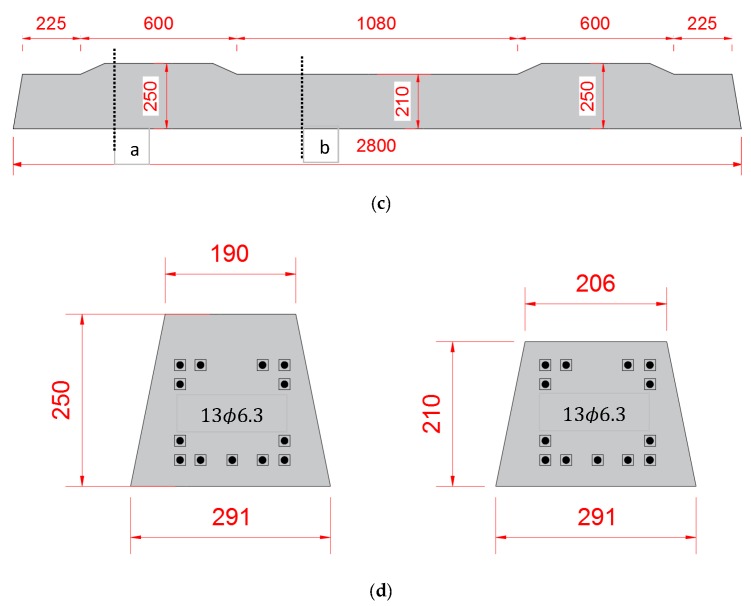
Detail of the geometry of the sleepers; (**a**) 3D view of the sleeper; (**b**) plan view; (**c**) side view; and (**d**) cross-section of the prestressed concrete sleeper.

The sleepers were designed for a positive moment of 2856 and 1786 kN.cm for the negative moment in the center of the sleeper. An initial prestressing force of 51 kN was applied to the 13 ϕ 6.3 mm wires. Figure 1 shows the geometric dimensions of the sleeper specimens. 

### 2.2. Compressive Strength Test of Concrete and Experimental Procedures

**Figure 2:**
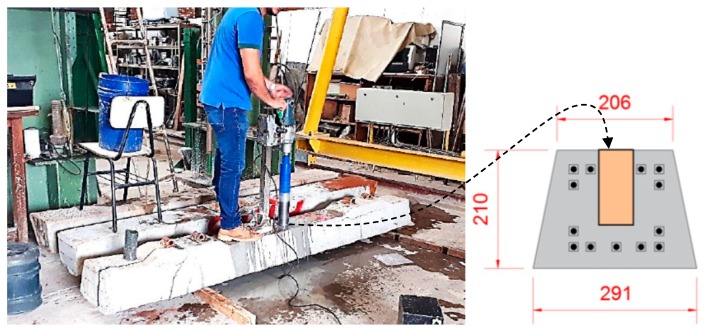
Execution of specimen extraction for concrete testing.

For the concrete compressive strength test, two specimens were extracted from five sleepers of the same batch, using a drill bit with a water-cooled diamond drill, which was capable of extracting specimens with a diameter of 68 mm and a height of up to 200 mm (see Figure 2).

After the extraction of the specimens, the surfaces at the end of the specimens were leveled with the aid of a grinding machine with a diamond disc, and then the compression test was carried out by using the procedure of the Brazilian standard ABNT NBR 5739 [24]; the results are shown in Table 1. After obtaining the results of the compressive strengths (*fc*), the analysis corrections indicated by Item 6.2 of the Brazilian standard ABNT NBR 7680 [25] were performed by using coefficients of correction *k*_1_*, k*_2_*, k*_3_ and k_4_, given by the following equation:

(1) $$f_{ci,ext}=\left[1+\left(k_{1}+k_{2}+k_{3}+k_{4}\right)\right]\cdot f_{ci,ext,initial}$$

where *k*_1_ represents a correction for the relationship between the height of the specimen (*h*) and the diameter (*d*); *k*_2_ would be the correction of the boring effect according to the diameter of the specimen; *k*_3_ is the direction of extraction in relation to the casting of the concrete, considered equal to zero because it was adopted that the direction of the concreting was parallel to the casting of the concrete; and *k*_4_ is the effect of moisture in the core; for structures without contact with water, this value is equal to 0.04, so it was possible to obtain the strengths of the concrete and the modulus of elasticity.

**Figure 3:**
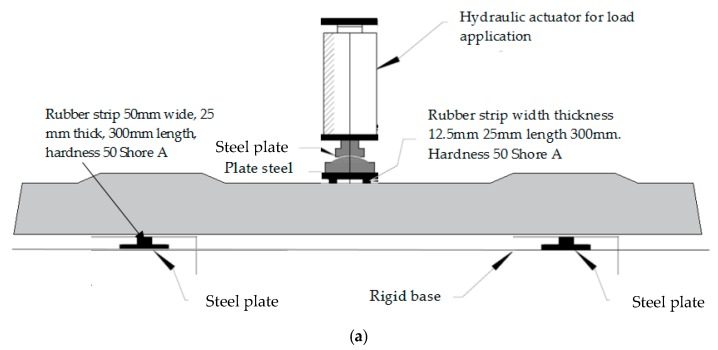

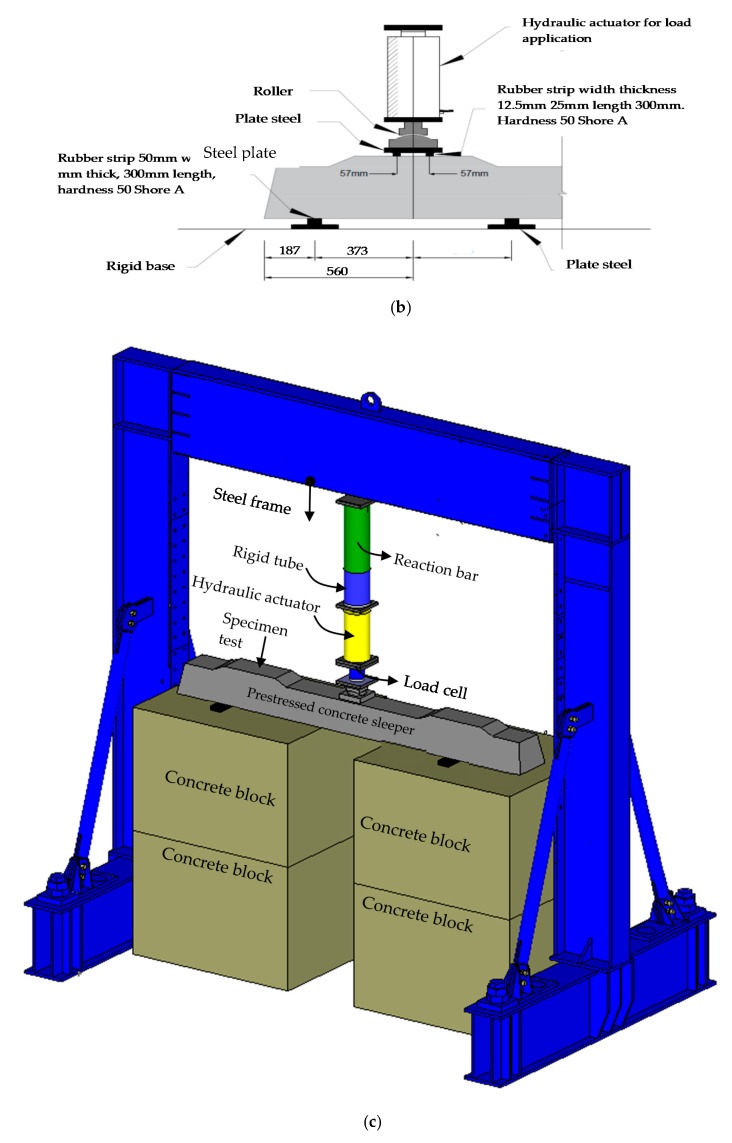

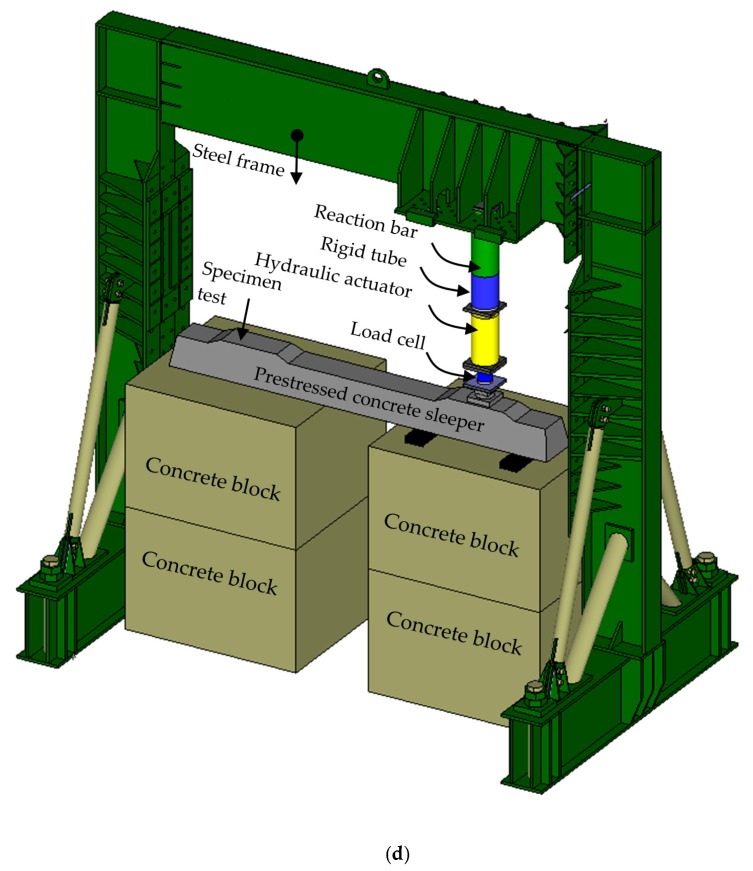
Experimental tests: (**a**) detail of positive moment test at midspan; (**b**) detail of positive moment test at the support; (**c**) positive moment test at midspan-schematic model in INFRALAB Laboratory; and (**d**) positive moment test at the support-schematic model in Structures Laboratory—LABEST.

Although the concrete strength adopted in the design is 50 MPa, it was found that condition of the concrete in the test age of about 1 year obtained an average strength of 63.67 MPa. The prestressing tendons are the chevron-patterned indented wires with ultimate strength of prestressed steel 1750 MPa. From visual inspection, it could be observed that the high-strength prestressing wires were of high quality, and thus the strength would not change so fast during the time. For carry out the experimental tests, the loading *P_j_* was applied at a speed of less than 50 kN/min, until the appearance of the first bending crack. Such moment generated with the first crack simulates the field conditions. Subsequently, a load was applied at a rate of 60 kN/min, until the sleeper failure. Figure 3 shows the details of the experimental test. 

**Figure 4:**
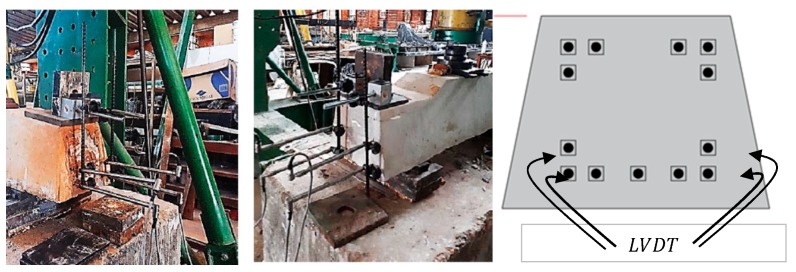
Position of wires tested with LVDT (Linear Variable Differential Transformer) to obtain the wire displacements, experimental test in Structures Laboratory.

Two test systems were developed; in the first test, eight sleepers were subjected to a positive moment test with load application at midspan of the sleeper supported on concrete blocks in the reaction frame. In the second, the load was applied on the base plate, which is attached to the rail of the railway, with ten experimental tests. In adherence tests, LVDT (Linear Variable Differential Transformer) was used to obtain the displacements at the load application point, and also in the prestressing wires at the end of the sleeper. Figure 4 shows details of the LVDT’s installed next to the prestressing wires in the sleeper.

For the simple bending test, this paper shows the details of the experimental tests by using one LVDT to measure the displacement in the center of the sleeper. The load was applied and controlled with a system set consisting of a three-phase motor responsible for pumping the hydraulic oil, a key that controlled the pressure outlet and a hydraulic actuator with a capacity of 1000 kN coupled in a load cell of 1500 kN. The reading was automatic by means of a system that recorded the data at a rate of 2 H_Z_. Subsequently, the results were plotted for comparison with a numerical model.

## 3. Experimental Results and Discussion

**Figure 5:**
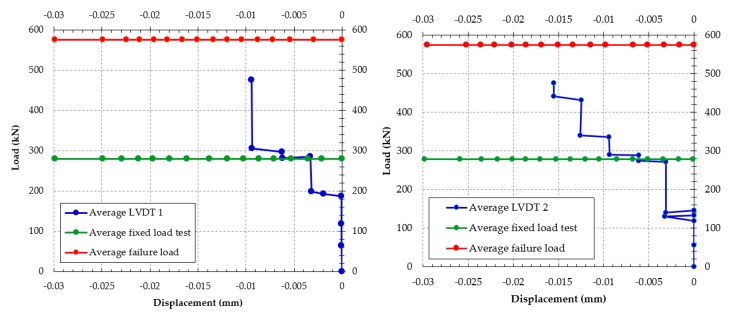

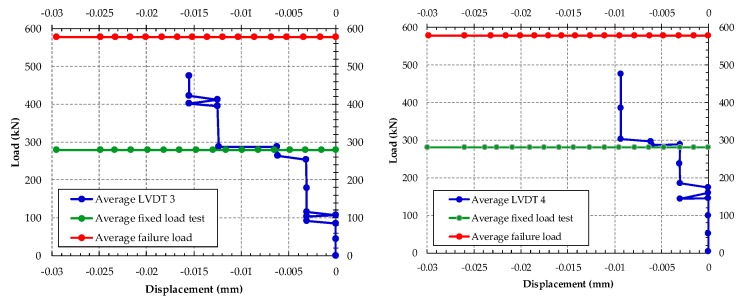
Average failure load of the sleepers tested with load on the support.

The sleepers with one year of use were removed from the railroad for testing. Ten sleepers were chosen to test the resistance in the rail support. In the load test of positive moment at the support, shown in Figure 4, an increasing load with a rate of 50 kN/min was applied, until reaching the total load of 278 kN, and kept for 8 min. The average slips of both the lower and the second layer threads were plotted in Figure 5. It can be seen that, during those 8 min, the wire displacements were less than 0.025 mm, in accordance with the allowed limit by the standard ABNT NBR 11709 (2015) [19]. Therefore, there is still good adherence between the concrete and the wires. After this, the load was increased until the sleeper failure. Table 2 shows the summary of the results of these tests.

**Figure 6:**
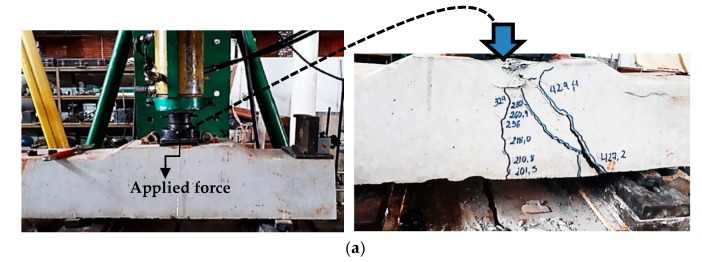

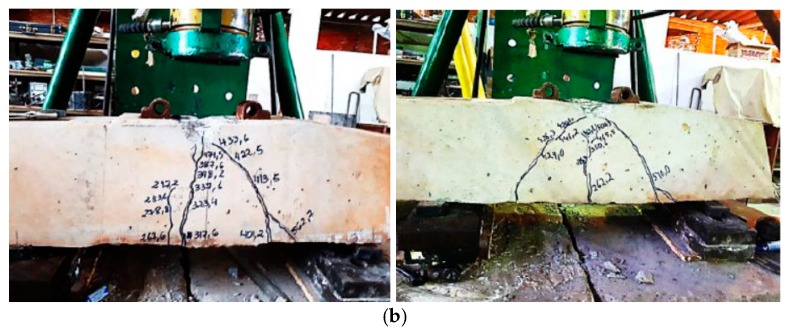
The main failure mode of the sleepers in the positive moment at support: (**a**) failure of the sleepers tested in the Structures Laboratory; and (**b**) failure by shear combined with flexion.

Figure 6 presents the main modes of rupture of the sleepers, with the load on the base plate of the rail.

In the positive moment test at the midspan with increasing load, the tensile failure stress was reached in the midsection, and the first vertical cracks with an average load of 278 kN appeared, covering half the height of the sleeper, or up to one just below the neutral line. In this phase, the tension forces were absorbed by the prestressed wires. After the beginning of this stage, the extreme sections of the concrete have not yet reached the breaking stress, but with the progressive increase in the load, the concrete began to have inclined cracks, until the failure of the lower wires. 

**Figure 7:**
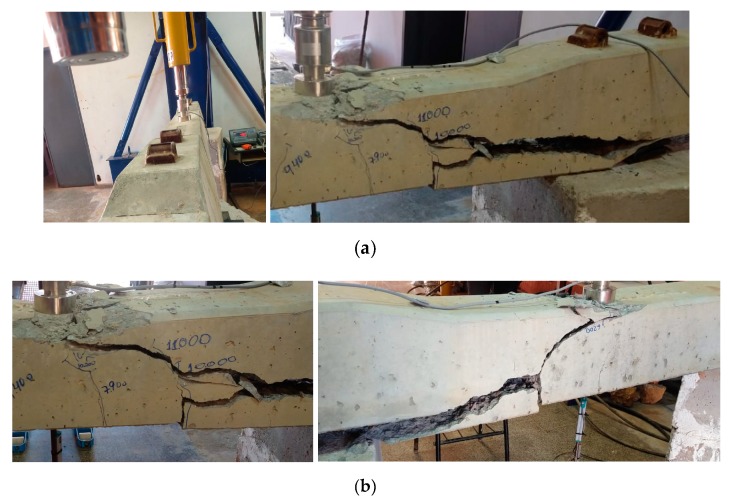

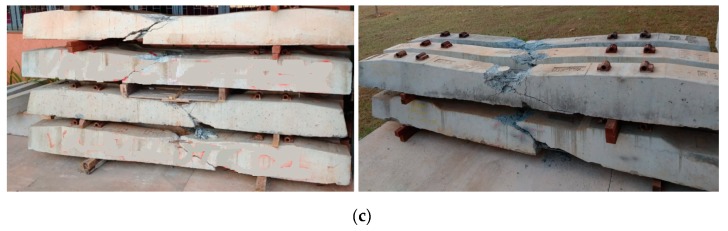
(**a**) Failure by simple bending; (**b**) failure in the positive moment test at the midspan; and (**c**) specimens tested in the INFRALAB Laboratory.

**Figure 8:**
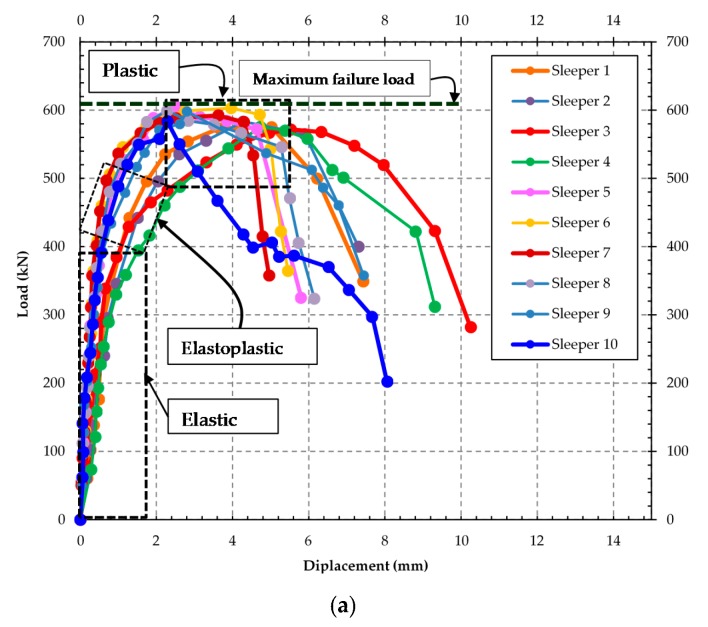

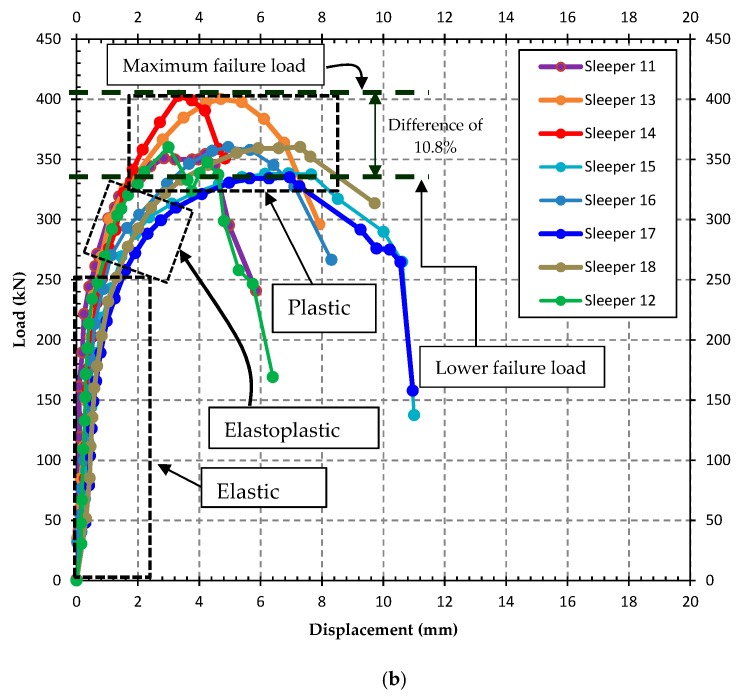
Experimental results: (**a**) positive moment test at midspan; (**b**) positive moment test at the support.

At this stage, the concrete showed crushing in the upper fibers, and the failure occurred with the increase of bending and shear cracks (see Figure 7). Figure 8 shows the load vs. displacement curves of the experimental tests.

As shown in Figure 8, the elastic stretch corresponds to loads of 400 and 250 kN, respectively. In this initial phase of the tests, the bending-moment values observed were not very high in the cross-section of the sleeper. The normal stresses at each point in the section had linear variation with the distance from the neutral line in the tension zone. Such zone stresses were lower than the tension strength of the concrete, already in the compressed zone, did not reach the compressive strength of concrete. Nevertheless, with the increase in the load values in the two tests with values of 500 and 350 kN (Figure 8a,b), the first cracks appeared, and the sleeper passed to have an elastoplastic stretch, and the concrete reached plastification in the tension zone; in other words, there is no linear response and the gradual increase of displacement.

In the first test, when the loads reached 566 kN, there was a total of plastification of the sleeper section with yielding of steel wire and concrete failure by crush of the compressed section. In the simple bending test, the plastic load that causes the failure of the sleeper was between 350 and 400 kN. At this stage of the test, the loss in tension strength of the concrete, the tension stresses were not completely resisted by steel wires, and consequently, it yields. Therefore, the failure range of the sleeper had a difference of 10.80% (Figure 8b). In all tests, it was evidenced the total use of the sleeper section with the equilibrium between the vectoral components of tension and compression.

## 4. Finite Element Analysis

### 4.1. Numerical Model of the Concrete Sleeper

In the concrete crack model, it is assumed that cracking is the most important factor in the material’s behavior. A “completely non-isotropic” behavior will govern the material after it has cracked. However, cracks influence the process of the computations and create problems for convergence of the results. 

Using the concrete crack model is advisable in cases where the concrete is under relatively low stresses (almost one-fourth or one-fifth of the pressure which could be tolerated by the concrete). 

Therefore, in this study, we used the Concrete Damage Plasticity (CDP) model, with ABAQUS [26]. The Concrete Damage Plasticity model uses the concept of isotropic damage in the linear region and combines the isotropic tensile strength and plasticity pressure, to show the nonlinear behavior of the concrete [27].The model introduced in this paper for the damaged concrete is a continuous model based with plastic behavior and the cracks caused by stresses are the main damage mechanism in the model [28].

**Figure 9:**
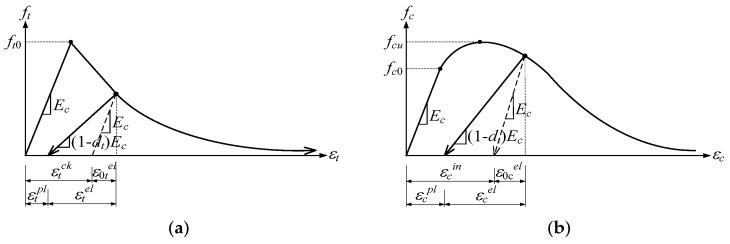
Concrete Damage Plasticity model: (**a**) tension behavior associated with tension stiffening; and (**b**) compressive behavior associated with compression hardening.

Damaged plasticity is assumed to characterize the uniaxial tensile and compressive response of concrete, as shown in Figure 9. At the beginning, the stress–strain relationship is linearly elastic under uniaxial tension, until the value of the failure stress, ft_0_, is reached. Failure stresses in concrete block are converted to replace microcracks in it. Beyond the state of the failure stress in concrete, stress–strain response is designed by softening characteristics (Figure 9a).

Under uniaxial compression, the response is linear until the value of initial yield, *f_c0_*. After attaining the ultimate stress, *f_cu_*, in the plastic zone, the response of concrete is characterized by the stress hardening, followed by strain softening (Figure 9b) [12]. 

Therefore, concrete stresses determined unloading from any point on the strain are given by the following equations:

(2) $$f_{t}=E_{c}\left(\varepsilon_{t}-\varepsilon_{t}^{pl}\right)\;\left(1-d_{t}\right)$$

(3) $$f_{c}=E_{c}\left(\varepsilon_{c}-\varepsilon_{c}^{pl}\right)\;\left(1-d_{c}\right)$$

where *E_c_* is the modulus of elasticity of concrete. Then, the effective tensile and compressive cohesion stresses of concrete are estimated as follows:

(4) $${\bar{f}}_{t}=\frac{f_{t}}{\left(1-d_{t}\right)}=E_{c}\left(\varepsilon_{t}-\varepsilon_{t}^{pl}\right)$$

(5) $$f_{c}=E_{c}\left(\varepsilon_{c}-\varepsilon_{c}^{pl}\right)\;\left(1-d_{c}\right)$$

which determine the size of the failure surface. The post-failure behavior of reinforced concrete represents by means of the post-failure stress as a function of cracking strain $\varepsilon_{t}{}^{ck}$ and $\varepsilon_{c}{}^{ck}$, which are defined as the total strain minus the elastic strain corresponding to the undamaged material, and tension stiffening data are given in terms of the cracking strains [27]. The implantation of the damage method with the plastic deformation was inserted in the ABAQUS program, where the user adds the data of the material properties obtained in experimental tests. ABAQUS automatically calculates the graph of the strain, using the low equations ABAQUS [29]:

(6) $$\varepsilon_{t}^{pl}=\varepsilon_{t}^{ck}-\frac{d_{t}}{\left(1-d_{t}\right)}\frac{f_{t}}{E_{0}}$$

(7) $$\varepsilon_{c}^{pl}=\varepsilon_{c}^{ck}-\frac{d_{c}}{\left(1-d_{c}\right)}\frac{f_{c}}{E_{0}}$$

where $\varepsilon_{t}{}^{ck}$ represents the plastic tensile strains of the concrete, and $\varepsilon_{c}{}^{ck}$ represents the plastic compression strains of the concrete. Property values for the assumed constitutive model of concrete and steel discussed above were collected from the specimens’ test results carried out in the INFRALAB Laboratory and the Structures Laboratory at the University of Brasilia.

### 4.2. Material Models

**Figure 10:**
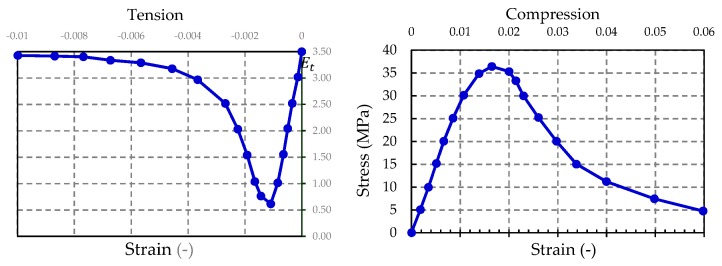
Physical laws of the concrete modeled in the numerical calculations.

In the FEM analysis, the Young’s modulus of concrete was adopted as *E*_0_ = 38 GPa, and the Poisson ratio as *v* = 0.17. By using Equations (4) to (7), it was possible for us to obtain the curves shown in Figure 10, which shows the parameters defining the nonlinear behavior of concrete, in both the tension and compression zones [30]. The physical laws of concrete were described by using the function proposed by [31].

This model includes combinations of non-associated plasticity with hardening and scalar isotropic elastic damage, to determine irreversible changes that occurred during the loading and unloading cycles. The CDP model is based on the brittle–plastic degradation model created by Lubliner et al. [32,33] and later perfected by Alfarah, López-Almansa and Oller [31] which assumes that the two main mechanisms of destruction are cracking as a result of stretching and crushing of the concrete under compression. In Table 3 presents the dimensionless properties of the concrete, using as input file in ABAQUS [34].

The concrete, in turn, was described by using the Abaqus Concrete Damage Plasticity model (Table 3), which is used for comprehensive modeling of concrete in a complex stress state [35,36,37,38,39,40,41,42].

The Concrete Damage Plasticity (CDP) model was used in the modeling of concrete, present in the ABAQUS material library and also the parameters in Lubliner et al. [32,33] and [42]. This constitutive model is suitable for materials that have different resistance to tension and compression; besides, it links the theory of plasticity with the mechanics of damage, being able to numerically simulate the degradation of the stiffness and failure of the concrete. The failure mechanisms considered are based on tension cracking and compression crushing. The CPM assumes a non-associative plastic flow rule, where the Drucker–Prager hyperbolic function is applied to define the potential flow. In this study, an elastic–plastic constitutive model was used to simulate prestressing steel.

This constitutive model is present in the ABAQUS material library, under the name PLASTIC. The PLASTIC model adopts the flow criterion of Von Mises, with an associative flow rule, ideal for the modeling of ductile materials such as steel.

**Figure 11:**
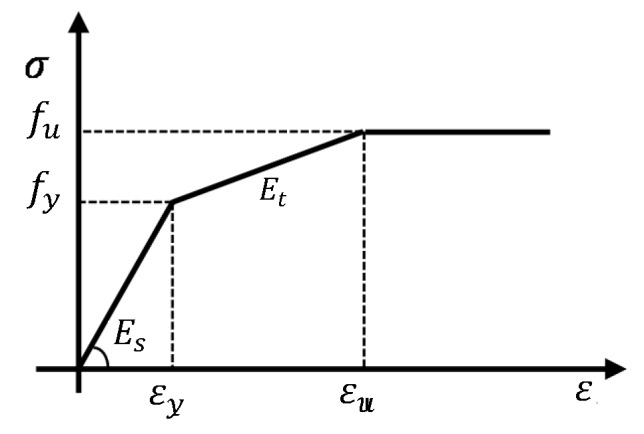
Stress–strain relationship for steel material.

The uniaxial behavior implemented in the model consisted of the bi-linear stress–strain [30,33]. Table 4 shows the details adopted for steel. The elastic–plastic material model was adopted for the constituent elements of the sleeper. Based on the yield criterion, the material was subject to the plastic flow rule and kinematic hardening rule (see Figure 11). Hardening was determined by adopting a tangent Young’s modulus of *E_t_ = 0.01E.*

The explicit dynamic analysis used in this paper was a strategy due to the greater possibility of numerical convergence, based on the nonlinear analysis method with time control. Despite being a dynamic analysis, this method is capable of performing quasi-static analyses, provided that small increments of loads are applied, so that the low effect of inertia prevails. It is very efficient in the analysis of complex numerical models that involve material damage, large deformations and contact interactions between components.

### 4.3. Loading Application and Boundary Conditions

**Figure 12:**
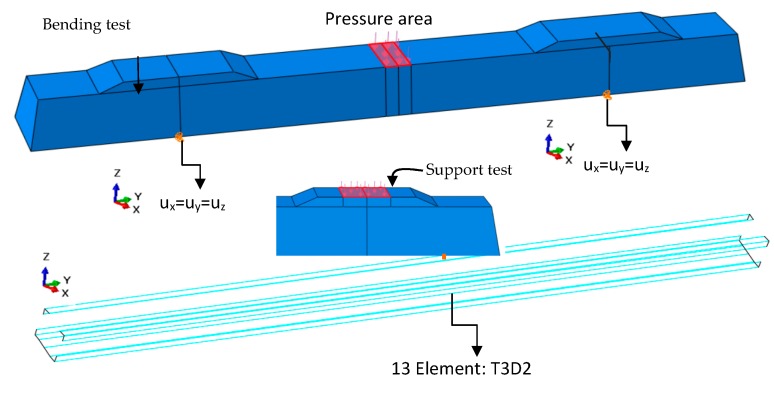

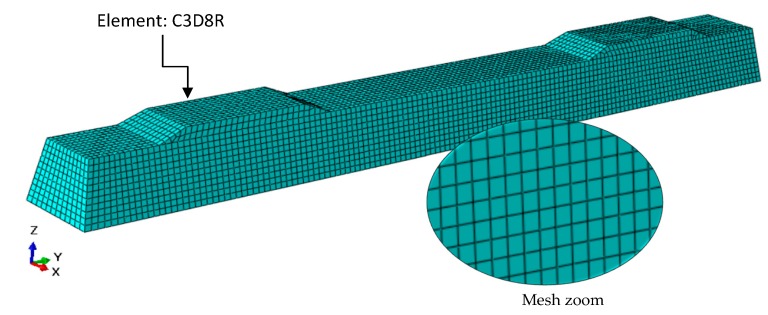
Boundary conditions adopted in the numerical model.

The loading points for external loads were selected for the standard one-point beam-bending configuration, in such a way that the bending moment was combined with shear (see Figure 12). In the numerical computations, the concentrated load was applied perpendicularly to the loaded surface, through many points, considering a perfect rigid body.

### 4.4. Validation of Numerical Models

The models were carried out by using eight-node reduced-integration solid elements (C3D8R—concrete sleeper) and two-node linear truss elements (T3D2—prestressed wires in concrete). Both were modeled discretely as elements embedded in the concrete of the sleeper. In all computations were considered both the physical nonlinearity of the materials and the geometric nonlinearity resulting from high deformation. The numerical models were verified based on the list of calculated displacements, ultimate loads and failure modes of the investigated sleepers and numerical model. For the simulation, we used the Riks [43] method, implemented in ABAQUS [25], with arc length and geometric nonlinearity [44].

**Figure 13:**
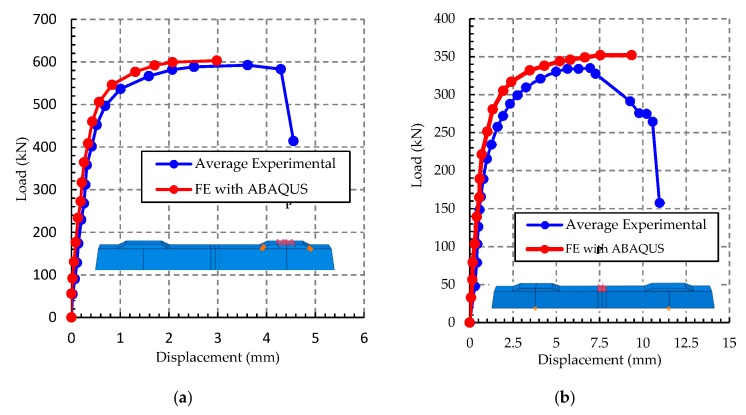
Comparison of vertical displacements (*U_z_*), determined through experimental tests and numerical analysis: (**a**) load at sleeper support; and (**b**) load at midspan.

The deflection used was in the vertical plane of the cross-section, at the middle of the span on the external part of the sleeper (*U_z_*). The highest displacements determined in the numerical analysis (*U_z_, FE*) were similar to the results of laboratory tests (*U_z_, Exp*). The largest difference between the numerical and experimental deflections) was 5% (see Figure 13).

For the extreme sections, under simple bending of the sleeper, the idealized model is similar to a truss, which considers the interaction between the bending moment and the shear force. In this model, the truss has longitudinal, compressed and tensioned flanges, as in the midsection, connected by top compression web (compressed concrete) and bottom chord. 

**Figure 14:**
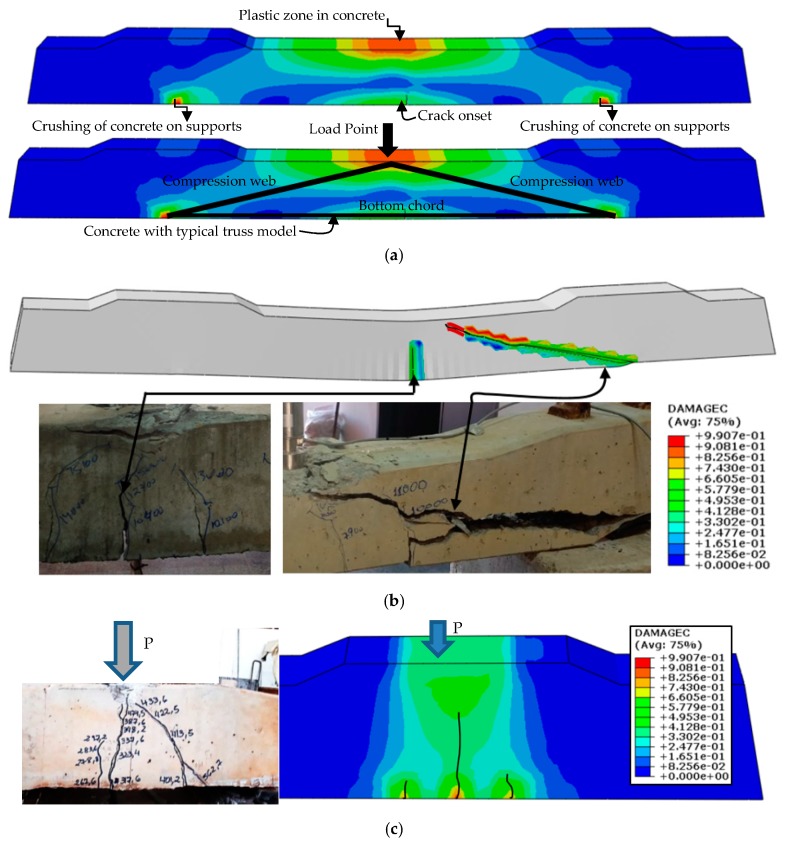
Comparison of failure modes: (**a**) idealization of the sleeper failure model to the plane truss; (**b**) comparison of failure modes of the bent sleeper; and (**c**) comparison of sleeper failure modes, with load applied at the support.

The compressed diagonals, called connecting rods, represent the concrete between the cracks. The bottom chord of the truss represents the prestressing wires. Figure 14 shows the failure modes of the sleepers.

## 5. Pathologies Simulation 

The first recurring problem that can result in structural failure in sleepers, especially in heavy-rail prestressed sleepers, is the failure by Rail Seat Abrasion (RSA). This deterioration is the result of an abrasion process, which is a gradual wear of the concrete cement paste, exposing the aggregate of the sleeper surface below rail foot [13,36,37]. RSA is related to weather, traffic and layout conditions. The wear of the concrete is due to the shear stress between the rail seat and the rail pad. Factors such as axle load, traffic volume, grid line curvature and inclination, presence of fines, water, and freeze-and-thaw cycles contribute to the wear of this region. Additionally, over the past two decades, durability and longevity of concrete structures have also been the focus of many researches. Amongst the main causes that lead to lifetime reduction is cracking due corrosion of the reinforcement wires prestressed.

**Figure 15:**
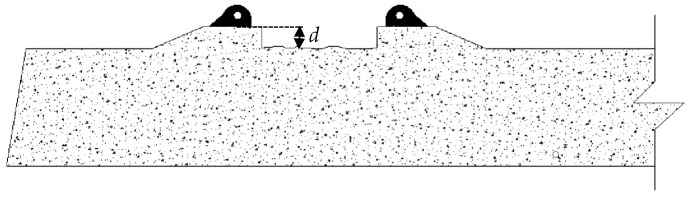
Detail of the Rail Seat Abrasion.

In order to analyze the RSA, the concrete abrasion of the sleeper was simulated in the ABAQUS program. Four damage cases of abrasion by reduction of depth (*d*) were applied (10, 20, 30 and 60 mm). The location and dimension of abrasion are shown in Figure 15, and reduction depth was simulated. This parametric study on ABAQUS consisted of decreasing the length of the entire wire along the length of the sleeper, to simplify the model. If the reduction of the diameter of the wire was located in a part of its length, the model would be very complex. It will require modeling the wires with Shell element, consequently, with the use of different types of nonlinear contacts; thus, the model was simplified.

**Figure 16:**
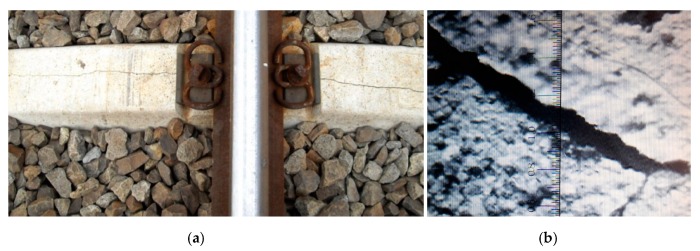
Detail of the cracked sleeper on the Brazilian railway: (**a**) sleeper with crack in the support; and (**b**) crack detail with laser fissurometer.

In order to simulate the pathology in the numerical model, we performed a reduction in the diameter of the protection wires. It was observed in practice that, on railway lines, sleepers in use suffer cracks next to the supports. The depth was obtained by inspection; the maximum crack value was 60 mm. Figure 16 shows details of a cracked sleeper. The value of the decrease in wire diameter was based on the literature [15,16,17].

**Figure 17:**
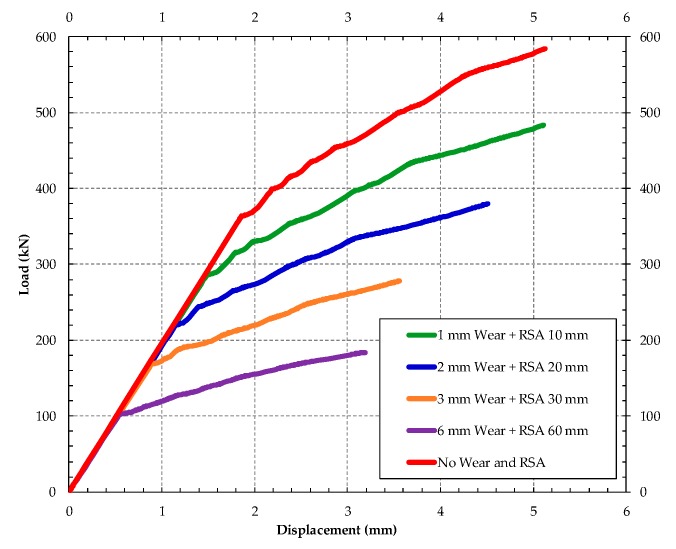
Failure modes for different lengths of pathologies in concrete of the sleeper.

In order to simulate the corrosion of the bars, four damage cases were applied by diameter reduction of the lower bars (1, 2, 3 and 6 mm), for failure with numerical simulation of the sleeper in ABAQUS [32]. The element T3D2 was used in the model for the prestressed wires, considering their adhesion with concrete made by the embedded region iteration that considers the bars perfectly anchored in the concrete. The combined effects of RSA and corrosion were simulated in ABAQUS. The load-deflection responses for the sleepers are shown in Figure 17. Observe that the increase of the abrasion depth reduces the stiffness of the sleeper. 

A decrease in the load capacity was expected, since the bending strength is provided mainly due to the active reinforcement located in the tension regions of the concrete; this fact is observed in the Figure 14. With a degradation of 1 mm in the diameter of the lower reinforcement and RSA of 10 mm, it was possible to notice a 16.52% reduction in the force to initiate the plastic behavior of the section (from 587 to 490 kN); for a degradation of 2 mm diameter and RSA of 20 mm, the active reinforcement had a reduction of 33.56% (390.28 kN); for 3 mm and RSA of 30 mm, a reduction of 49.53% (290.78 kN), while a total corrosion and RSA of 60 mm, and a reduction of the load capacity in a 68.92% (182.42 kN).

## 6. Conclusions

This paper presents the experimental results of positive moment tests of prestressing sleepers and numerical results of finite analysis to investigate the static behavior of the sleepers. Based on the results, the following conclusions can be drawn in this paper:

- The experimental results pointed out that the sleepers were designed in accordance with Brazilian standards.
- The use of the CDP model for concrete behavior was considered satisfactory. The numerical mechanical behavior was similar to experimental ones from the beginning of plastification up to the final failure stage.
- Simulations of the post-buckling behavior of the structure (in post-critical states) were carried out, using the Riks analysis available in the Abaqus software [26], which enables “passing” through the bifurcation point. In this way, it was possible not only increase but also decrease the force in order to meet the static equilibrium criterion.
- The size of the calculation step in the modified Riks method [43] depends on the “Arc Length”, measured along the static equilibrium path in the load–displacement space.
- It was also possible to evaluate the impact of local pathologies in the sleeper’s load-carrying capacity. These deleterious effects of reinforcement degradation are responsible for reducing the sleeper strength, which may lead to a loss of more than 50% of the load-carrying capacity.
- It is important to highlight the relevance of prestressing, good construction practices (well-performed cure and low water/cement ratio) and other anti-cracking mechanisms in the durability of the sleeper in order to get a good response from the sleepers over their service life.
- The numerical model carried out could be used to predict static responses of prestressed concrete sleepers.

## Figures and Tables

**Table 1:** Results of the concrete extraction and compression test.

Sleeper Specimen	*d* (mm)	*h* (mm)	*h/d* (--)	*k_1_* (--)	*k_2_* (--)	*k*_3_ (--)	*k_4_* (--)	Area (mm²)	Load (kN)	Stress (MPa)
CP1	68	121.1	1.78	0.01	0.12	0	0.04	3631.7	79.4	85.0
CP2	68	121.0	1.78	0.01	0.12	0	0.04	3631.7	64.6	69.1
CP3	68	132.4	1.95	0.01	0.12	0	0.04	3631.7	57.7	61.7
CP4	68	132.9	1.96	0.01	0.12	0	0.04	3631.7	58.1	62.1
CP5	68	120.6	1.77	0.01	0.12	0	0.04	3631.7	49.2	52.6
CP6	68	110.7	1.63	0.02	0.12	0	0.04	3631.7	59.3	62.9
CP7	68	132.4	1.95	0.01	0.12	0	0.04	3631.7	57.7	61.7
CP8	68	132.9	1.96	0.01	0.12	0	0.04	3631.7	58.1	62.1
CP9	68	120.6	1.77	0.01	0.12	0	0.04	3631.7	49.2	52.6
CP10	68	110.7	1.63	0.02	0.12	0	0.04	3631.7	59.3	62.9

**Table 2:** Summary of positive moment at the support.

Sleeper Specimen	Reference Load (kN)	Average Load Applied (kN)	Load Time Applied (min)	Crack Load of the Sleeper (kN)	Failure Load (kN)
SS1	278.24	278.24	8	267.60	576
SS2	278.24	278.31	8	233.10	566
SS3	278.24	278.02	8	210.50	490
SS4	278.24	278.21	8	262.20	578
SS5	278.24	278.22	8	254.4	601
SS6	278.24	278.24	8	248.1	608
SS7	278.24	278.27	8	234.41	589
SS8	278.24	278.18	8	241.17	589
SS9	278.24	278.31	8	238.11	583
SS10	278.24	278.26	8	238.10	597

**Table 3:** Parameters of the Concrete Damage Plasticity model.

Internal Friction Angle β	Eccentricity of the Plastic Potential ϵ	*f*b0*/f*c0	Shape of the Plastic Potential Surface Kc	Viscosity Parameter μ
36°	0.1	*1.16*	0.667	0.005

**Table 4:** Material properties of steel in the numerical model.

Elastic Modulus, *E_s_* (MPa)	Hardening Modulus *E_t_* (MPa)	Yield Stress, *f_y_* (MPa)	Poison Coefficient, ν (-)	Density (kN/m^3^)
205.000 MPa	200 MPa	1700 MPa	0.3	78
